# Domestic

**Published:** 1840-09-19

**Authors:** 


### DOMESTIC.

*Cases of Operations for Procidentia Uteri.* By E. Geddings, M. D., Prof. of Pathology and Medical Jurisprudence in the Medical College of the State of South Carolina.—*Case* I.—July 8th, 1839, Betty, a female slave, ætat. 35, the propery of J. H. R., Esq., was placed under my care, on account of procidentia uteri. She is the mother of one child, but has been affected with her distressing infirmity for upwards of fifteen years, to such a degree, that the uterus protruded from the vulva in form of a large ovoid tumour, equalling in size the head of a mature fœtus, and carrying with it the inverted vagina. From long exposure, the mucous membrane had become rough, thickened, and scaly; the tumour hung pendulous between the thighs, where it was exposed to constant friction, and excoriation, which, together with the constant dragging pains which she experienced, disqualified her for every kind of exertion, and reduced her to a state of perpetual suffering. As the prolapsed organ could be easily replaced, but not retained, all the ordinary mechanical means having previously proved unavailing, I resolved to perform the operation of *episioraphy*, recommended, and successfully practised by Fricke, of Hamburg. The vagina, however, being greatly relaxed, and very capacious, I deemed it advisable so to modify the operative procedure, as to combine with it the advantages of the plans proposed by Marshall Hall and Professor Dieffenbach.

The operation was accordingly performed in the following manner, in presence of several of my medical friends, who were present on the occasion. The hair having been removed, the patient was placed in the ordinary position for lithotomy, and the prolapsus reduced; seizing one labium with the left hand, while it was put upon the stretch by an assistant, an incision was commenced, with a common scalpel, about a finger's breadth from the upper commissure and the same distance from the edge of the labium. This was carried downwards with a bold sweep, and terminated by a slight curve inwards, at a little distance behind the fourchette. A slip of the labium, a finger's breadth in thickness, was thus severed from the external parts, taking care not to cut through the mucous membrane of the vagina. Making traction on this slip, downwards and inwards, the mucous membrane of the lateral portion of the vagina was then dissected up to the extent of an inch and a half, and detached with the excised labium. The same thing was repeated on the opposite side, the incision being so directed, as to intersect the first cut at an acute angle, and remove the fourchette with the other parts. After the hæmorrhage, which was inconsiderable, had ceased, an oiled sponge was passed into the vagina, and the two raw surfaces were brought in apposition, by means of the quilled suture of five stitches, the first inserted near the inferior angle. A catheter was placed in the bladder; the parts were dressed with a compress of soft lint, secured by a T bandage, and the patient was put to bed, with her thighs and ankles properly secured together by means of bandages.

On the subsequent day, it was found that the urine escaped continually by the side of the catheter. This was consequently removed, and as the patient experienced no inconvenience in passing urine, it was not again introduced. Simple dressings were applied until the fourth day, when, union having taken place, the sutures were removed. From this time forward, the case continued to proceed favourably. On the sixth day the sponge was withdrawn, and on the tenth, menstruation took place and continued for the usual period without deranging the healing process in the slightest degree. In about a fortnight, firm union had taken place throughout the whole length of the wound, except to the extent of about three quarters of an inch near the inferior angle. This point was touched, at each dressing, with a solution of sulphate of zinc, under which treatment it closed in a few days, and by the end of the third week, my patient was able to walk about. She was, however, suffered to remain in the city for some weeks afterwards, during which time she suffered no inconvenience, except occasional slight uneasiness about the pelvis, dependent, no doubt, upon the pressure of the engorged and indurated uterus upon the walls of the vagina. She was finally sent by her owner to his plantation near Georgetown, apparently delighted with her new situation.

Dr. B. having read a brief notice of the preceding case, which, with some others, was published from a private letter, by my friend Professor Dunglison, in his Medical Intelligencer,* was induced to send me the next two cases, under the hope that they might be relieved by the same procedure. It may be proper to state, that they had both been perseveringly treated for some time, with various pessaries, Hull's utero-abdominal supporter, &c., without the effect of overcoming their infirmity.

*Case* II.—Mary, a female slave, aged about thirty, of spare frame, the mother of —— children, has been for a long time affected with procidentia uteri. The tumour, an ovoid figure, and as large as the head of a child, is hard, rough, and scaly upon the surface. On the right side, there is an ulcerated surface, with hard edges, of an inch, or more, in diameter, occasioned by the friction against the right labium and thigh; the whole vagina being inverted, and the protruded organ hanging pendulous from the vulva.

*January* 14*th*, 1840. In presence of the class of the Medical College of the State of South Carolina, and several of my medical friends, I returned the uterus, and performed the operation of episioraphy, precisely as in the preceding case, omitting to leave the catheter in the bladder. In the afternoon, the patient complained of considerable pain over the region of the bladder and uterus, and suppression of urine. Hot steeps were directed to be laid upon the lower part of the abdomen; thirty drops of laudanum to be given. The suppression and pain soon disappeared; and but little inconvenience was experienced at any future period. On the fifth day, one of the middle sutures was removed, union having already taken place, and at the next dressing all were withdrawn. On the eighth, the sponge was taken from the vagina; in a little more than a fortnight, my patient was able to walk about the room; and at the end of the third week, she was sent home, the wound being nearly all cicatrized, and the uterus being apparently well supported in its proper situation.

I should state, that in the progress of this case, I felt considerable apprehension on account of a violent cough, with which the patient was affected, fearing that the efforts of coughing must force the uterus downwards with such violence as to turn out the sutures. This was soon relieved, however, by syrup. scill. comp. c. tinct. opii camph.

*Case* III.—Maria, a female slave, aged about twenty-eight, the mother of —— children, also the property of Dr. B——, is affected with procidentia uteri, which presents the same characters and appearances of the preceding case. The ulceration, however, is less extensive, and the tumour is not quite as large.

I operated on this woman at the same time and place with the preceding. This operation was performed in the same manner, and precisely the same treatment was pursued. For a few days, the wound did not appear to cicatrize quite so favourably as in Mary's case, and she suffered considerably from pain in the back, owing, probably, to position. She was sent home at the end of the third week, having been able to walk about, without inconvenience, for some days previously.

*Case* IV.—April, 1840. A young female slave, the property of Mr. T. M. H——, is affected with procidentia uteri, with complete inversion of the vagina, and has elephantiasis of one leg, extending as high as the knee. She has never borne children, and can give no account of the cause or first origin of her infirmity, with which she has been affected for several years.

I performed the operation of episioraphy, in presence of my pupils, in the same manner as in the cases detailed above, merely omitting to leave a sponge in the vagina, and using only four sutures to close the wound. At the fourth dressing, I found the space between my pa-

* Vol. III. p. 145. 1839.

tient's thighs, as low as the knees, completely flooded with dark, putrid, excessively fœtid blood, and a large quantity of the same fluid, escaped from the vagina, on making pressure over the pubes. This at first gave me some uneasiness, but as it ceased to come away, I attributed it to a gradual oozing from the denuded walls of the vagina, taking place after the parts had been closed by suture. Had I inserted the sponge as in the preceding cases, this would probably have been obviated.

On the fifth day, the sutures were removed, and, in three weeks, the parts were completely cicatrized. During nearly the whole course of treatment in this case, violent paroxysms of pain, apparently of a nervous character, were experienced at night, requiring for their relief, large doses of laudanum.

I this day, June 18th, visited the subject of the last case, with the view of ascertaining whether the success of the operation was likely to prove permanent. I was gratified to find her in excellent health: improved in *embonpoint* and in her general appearance, and completely relieved of her infirmity; having at no time since her recovery, experienced the slighest tendency to, or return of, the prolapsus, nor does she suffer any pain, or other distress, about the region of the uterus. Magister ejus mihi dixit, ita libidinosam, ut fere omni nocte, assidue conquirit commercium hominibus, ut nil dubitat sæpenumero congressu venere fruitur.

I also called this morning, on Mr. R——, the owner of the subject of case first. Not being so fortunate as to find him at home, I made particular inquiry of his coachman, who had just returned from Mr. R——'s plantation near Georgetown, relative to the patient's present condition. I learned from him that she is in good health and spirits, free from her former infirmity, and fully able to do the labour imposed on her. Of the other two cases, I regret that I cannot speak with such certainty, as they both reside in the country; but I entertain no doubts of the result being equally favourable with them, as it has proved in the first and fourth individuals.

It now only remains for me to make a few observations, relative to the comparative advantages of the different procedures which have been described above.

The actual cautery and the various caustics which have been used to fulfil the same indication, are liable to some serious objections. Setting aside the pain resulting from their application, which I think must be almost insupportable, notwithstanding the assurances of their advocates to the contrary, there are probably but few females who would be disposed for any consideration short of death itself, to have the cavity of the vagina approached with a red hot poker, or, what is scarcely less appalling, the fuming nitric acid. Nor can the extent of the action of these agents be limited with any certainty; and it must be borne in mind, that there is great danger of the development of metritis, cystitis, and peritonitis, from this free cauterization of the vagina. Add to all these the still more important one, that the operation must often fail, (I believe this would happen in every case similar to those described above,) and we have a strong argument against the use of cautery in any of its forms.

The operation of Kolporaphy (Marshall Hall and Dieffenbach) does not expose the patient to so much danger as that by cautery, but I am disposed to believe, that in a majority of cases it will fail of ultimate success. It is true, the advantages seem to have been permanent in Dr. Hall's case, and the same may be true of some of the others, but it is notorious, that in a large proportion of the cases reported by Berard, it either failed from the first, or the prolapsus returned after the individuals had been reported as cured. Besides, should impregnation take place, with a vagina contracted through its whole length, to the extent it must be, to keep up a prolapsed uterus, it is obvious the child could not pass without previously enlarging the canal by free incisions; an operation that could not be performed without considerable difficulty.

When, therefore, the whole grounds are impartially considered, there cannot be the slightest hesitation in awarding the preference to Fricke's operation. It is attended with comparatively little pain; is for the most part free from danger; and rarely fails to prove completely successful—Fricke having cured three out of four, and all my cases, four in number, having terminated favourably. Should the operation fail, it can be repeated, and possesses this important advantage that it can be performed at any period of life, as it does not interfere with menstruation, conception, or child bearing. My fourth case proves that it does not of necessity impose upon the female the restraint of living *absque marito*, and Plath gives an account of a female operated on by Fricke, who conceived, and brought forth a child, notwithstanding the barrier at the orifice of the vagina.* Should this create an obstacle, it could be removed in an instant by a simple nick of the scalpel.

I do not write, however to decry either operation. My object has been, to collect together the amount of our information on the subject; to add to it the results of my own experience; and thus to put within the reach of a large number of my professional brethren, who, from their retired situation, have not easy access to the current medical literature of the day, a means of relieving an infirmity, which, from its frequency, especially amongst the plantation slaves of the southern states, must fall very often under their observation.

---

* Zeitschrift fur die gesammte Medicin. Bd. ii. p. 142.

